# A self-aggregating peptide: implications for the development of thermostable vaccine candidates

**DOI:** 10.1186/s12896-019-0592-9

**Published:** 2020-01-21

**Authors:** Adolfo Cruz-Reséndiz, Jesús Zepeda-Cervantes, Alicia Sampieri, Carlos Bastián-Eugenio, Gonzalo Acero, J. Iván Sánchez-Betancourt, Goar Gevorkian, Luis Vaca

**Affiliations:** 1 Instituto de Fisiología Celular, Universidad Nacional Autónoma de México, CDMX 04510 Mexico City, Mexico; 2 Instituto de Investigaciones Biomédicas, Universidad Nacional Autónoma de México, CDMX 04510 Mexico City, Mexico; 3 Facultad de Medicina Veterinaria y Zootecnia, Universidad Nacional Autónoma de México, CDMX 04510 Mexico City, Mexico; 4 Department of Physiology and Biophysics, University of Washington School of Medicine, Seattle, WA 98124 USA

**Keywords:** Vaccines, Particles, Self-assembling, Thermostable, Immunology

## Abstract

**Background:**

The use of biomaterials has been expanded to improve the characteristics of vaccines. Recently we have identified that the peptide *PH*_*(1–110)*_ from polyhedrin self-aggregates and incorporates foreign proteins to form particles. We have proposed that this peptide can be used as an antigen carrying system for vaccines. However, the immune response generated by the antigen fused to the peptide has not been fully characterized. In addition, the adjuvant effect and thermostability of the particles has not been evaluated.

**Results:**

In the present study we demonstrate the use of a system developed to generate nano and microparticles carrying as a fusion protein peptides or proteins of interest to be used as vaccines. These particles are purified easily by centrifugation. Immunization of animals with the particles in the absence of adjuvant result in a robust and long-lasting immune response. Proteins contained inside the particles are maintained for over 1 year at ambient temperature, preserving their immunological properties.

**Conclusion:**

The rapid and efficient production of the particles in addition to the robust immune response they generate position this system as an excellent method for the rapid response against emerging diseases. The thermostability conferred by the particle system facilitates the distribution of the vaccines in developing countries or areas with no electricity.

## Background

Vaccines are considered one of the most important medical advances in the history of humanity, preventing and eradicating diseases [1, 2]. The World Health Organization (WHO) estimates that vaccines save around 2–3 million lives a year [3]. Traditional vaccines are based on two main methodologies: live-attenuated and inactivated/killed pathogens [4]. Even though vaccines produced with these methods are immunologically effective, they still show some disadvantages, such as the need for a cold-chain, reduced shelf life and the time-consuming processes involved in the production and purification [5–7]. On the other hand, new methodologies have been used such as subunit and recombinant vaccines that weakly stimulate the immune system and their immunological effect is of short durability, so they require the use of adjuvant to potentiate their effect. Currently available adjuvants may lead to unwanted effects such as the generation of granulomas, allergies and neurotoxicity due to the different components used [5, 8, 9]. However, even with the evolution of vaccines, vaccination continues to represent a high cost mainly for developing countries, due to the fact that they have the highest number of people vulnerable to infectious diseases [10–12]. An effective, low cost technology to produce thermostable vaccines would represent a major advancement in the fight against infectious diseases worldwide, and may significantly reduce the risk of pandemics [13].

For this reason, in recent years new technologies have been developed to advance in the production of more efficient and safer vaccines [10, 14, 15]. Lowering the cost of vaccines is an essential step to facilitate massive vaccination especially in isolated areas where the cold-chain cannot be maintained easily [14, 16]. This last point is especially important since the cold chain represents about 80% of the cost of vaccines [17, 18].

The use of biomaterials are a central part of novel strategies to develop next-generation vaccines [19, 20], delivery systems [21, 22] with improved thermostability [23].

Some insect viruses have developed a remarkable strategy to maintain virus viability for years at ambient temperature. The strategy is based on the generation of crystal structures known as polyhedra, where the virus is occluded and protected from the environment for several years. Most interestingly, a single protein (known as polyhedrin) forms the polyhedra crystal during the infection of insect cells. Polyhedrin self-aggregates inside the nucleus and during aggregation viruses get occluded inside the crystal. Thus polyhedra is a natural preservative of proteins, whose function is to maintain the virus viable for many years at ambient temperature [24, 25].

One of the most studied insect viruses that forms polyhedra is the *Autographa californica multiple nucleopolyhedrovirus* (AcMNPV) [26, 27].

We have recently identified an amino acid sequence in the polyhedrin protein from AcMNPV, which maintain the self-aggregating properties of the full-length protein [28]. This sequence includes the first 110 amino acids from polyhedrin (*PH*_*(1–110)*_). We have shown also that *PH*_*(1–110)*_ self-aggregates even when other proteins or peptides are fused to its sequence. Furthermore, we have recently shown that the ORF2 from porcine circovirus (PCV2) fused to *PH*_*(1–110)*_ injected in pigs results in the generation of neutralizing antibodies against circovirus [29]. However, no characterization of the particles formed or the thermostability of the vaccine and the adjuvant properties conferred by *PH*_*(1–110)*_ were analyzed in the aforementioned study [29].

In the present study we fused the green fluorescent protein (GFP) to the *PH*_*(1–110)*_ sequence to produce a fusion recombinant protein that self-aggregates. The use of GFP facilitated the characterization of the particles using confocal microscopy. We used this fusion protein to characterize the formation of nano and microparticles and to explore its thermostability for several months as well as their capacity to generate antibodies when immunized in mice.

The results obtained show that the particles formed by *PH*_*(1–110)*_ preserve the function of the protein contained within for at least 1 year at ambient temperature. The particles formed by *PH*_*(1–110)*_ generate a robust immune response raising antibodies that recognize GFP. The particles showed adjuvant properties, since no adjuvant was required to generate a robust immune response against the antigen (GFP). The *PH*_*(1–110)*_ particles are easily purified by centrifugation, reducing significantly the cost of purification. All these results position *PH*_*(1–110)*_ as a novel platform for the production of thermostable vaccines contained inside nano and microparticles.

## Results

### *PH*_*(1–110)*_ peptide fused to GFP form *particles*

**Fig. 1:**
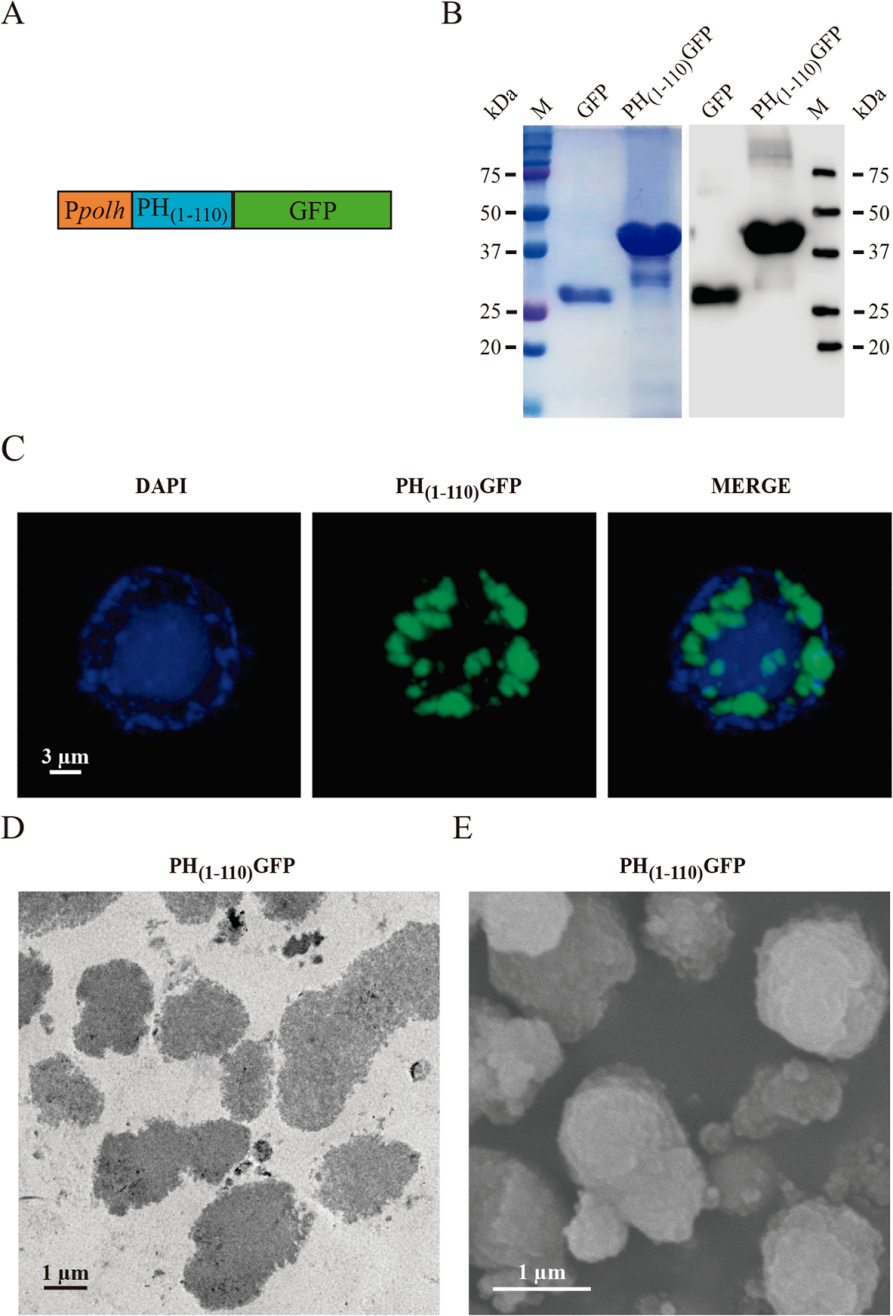
*Characterization of the PH*_*(1–110)*_ *GFP particles.* **a** Scheme of the genetic construct for the generation of recombinant baculovirus expressing *PH*_*(1–110)*_ *GFP particles*, in the orange box is shown the polyhedrin promoter (*polh)*, the blue box represents the 110 amino acids of the polyhedrin and the green box represents the GFP protein bound at the carboxyl terminus of polyhedrin. **b** SDS-PAGE (left) and WB (right) showing the bands of GFP protein expression (~ 28 kDa) and the *PH*_*(1–110)*_ *GFP* particles (~ 42 kDa). **c** In confocal microscopy image is observed in blue (DAPI) the nucleus of an insect cell (SF9) that contains inside the *PH*_*(1–110)*_ *GFP particles* (green). **d** TEM image of *PH*_*(1–110)*_ *GFP particles*, the particles are observed compact and irregular. **e** SEM image showing *PH*_*(1–110)*_ *GFP particles* of size different and irregular morphology

We developed a universal system to produce fusion proteins using as template the first 110 amino acids from AcMNPV polyhedrin protein. A transfer plasmid containing the strong polyhedrin promoter drives the expression of the *PH*_*(1–110)*_ followed by a poly-linker [29], which allows the insertion of any sequence to generate the fusion protein (Fig. 1a). In this particular case we introduced the sequence from the Green Fluorescent Protein (GFP) to produce the fusion protein *PH*_*(1–110)*_ *GFP* [28]*.* This plasmid was utilized to produce recombinant baculovirus expressing the fusion protein in Sf9 insect cells. Sf9 insect cells infected with our recombinant baculovirus carrying the gene to expressed the fusion protein *PH*_*(1–110)*_ *GFP* were sonicated to release the particles. Particles were centrifuged at low speed and the protein purified in this manner was subjected to SDS-PAGE analysis (Fig. 1b). As control we utilized a pure soluble form of GFP. As illustrated in the figure, a simple centrifugation results in highly pure *PH*_*(1–110)*_ *GFP* protein, showing that the major component is the expected protein*.* Figure 1c illustrates an example of a Sf9 insect cells expressing *PH*_*(1–110)*_ *GFP* visualized by confocal microscopy (for a 3D reconstruction of the particles please refer to Additional file 1: Video S1)*.* Notice that all particles were contained within the nucleus (labeled with DAPI in blue). Electron microscopy (transmission electron microscopy in panel D and scanning electron microscopy in E) shows that the *PH*_*(1–110)*_ *GFP particles* are polydisperse, formed micro and nanoparticles. A capillary electroforesis analysis indicates that over 80% of the protein content is *PH*_*(1–110)*_ *GFP* (see the Additional file 2).

### Fusion protein have slow diffusion inside *PH*_*(1–110*)_*particles*

To determine the rigidity of the *PH*_*(1–110)*_ *GFP particles*, we conducted fluorescence recovery after photobleaching (FRAP) studies using confocal microscopy. The idea behind this study was that in a crystal structure the *PH*_*(1–110)*_ *GFP* protein should have no diffusion, since a crystal lattice is rigid, whereas in a less rigid structure some diffusion should be observed. The speed of diffusion should be related to the laxity of the structure [30]. To conduct this, experiment a region of interest (ROI) in each of the particles was photobleached to eliminate the fluorescence of GFP in the ROI. Fluorescence recovery inside the ROI was monitored for several hours.

**Fig. 2:**
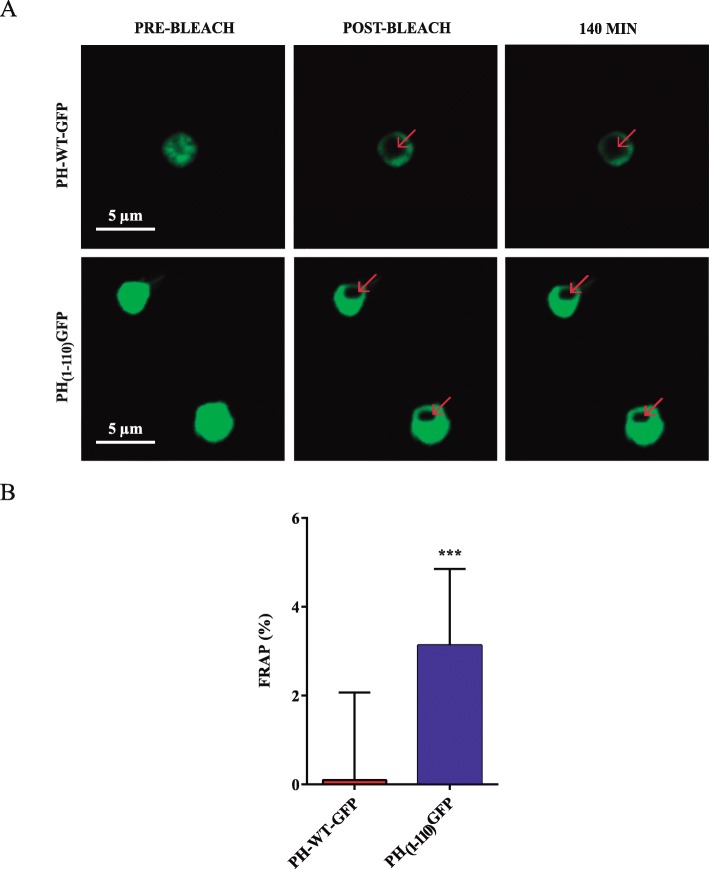
*PH*_*(1–110)*_ *GFP particles show lower rigidity than PH-WT-GFP particles.* **a** The images show the process of FRAP in *PH-WT-GFP* particles (top) and *PH*_*(1–110)*_ *GFP particles* (bottom). The bleach site is shown with red arrows and the panels on the right (140 min) show the last FRAP evaluation point. **b** Percentage of fluorescence recovery after 140 min of bleach. Error bars indicate the means ± SD; *n* = 10 for *PH-WT-GFP* particles; *n* = 14 for *PH*_*(1–110)*_ *GFP particles.* *** *p* < 0.001 (two-tailed Student’s t-test)

Experiments were conducted with wild type polyhedrin fused to GFP (*PH-WT-GFP)* and particles formed by *PH*_*(1–110)*_ *GFP* (Fig. 2a). Fluorescence recovery was followed for 140 min. As expected, the particles formed by *PH-WT-GFP* showed no recovery after photobleaching, indicating the lack of mobility of the GFP inside the crystal. Most interestingly, the *PH*_*(1–110)*_ *GFP* showed a partial recovery of fluorescence after 140 min. The time course of recovery after photobleaching is illustrated in Fig. 2b-c and quantification of recovery in Fig. 2d. *PH*_*(1–110)*_ *GFP* particles showed around 5% recovery after FRAP within the first 140 min, indicating a very slow diffusion of several hours.

### *PH*_*(1–110)*_*particles* are purified with a single centrifugation

**Fig. 3:**
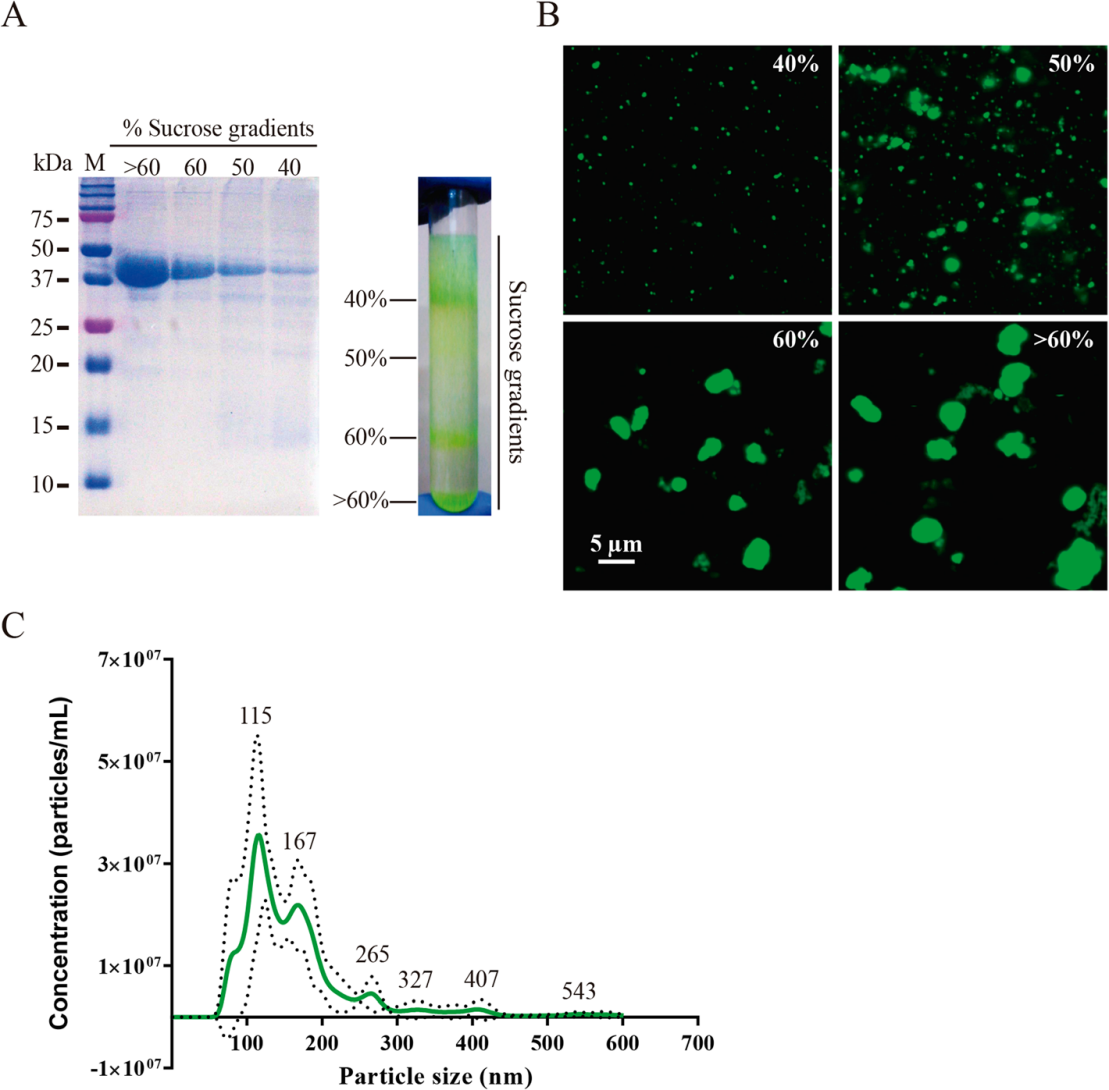
*The PH*_*(1–110)*_ *GFP particles are purified and separated by size in a single centrifugation step.* **a** Sucrose gradients (right) in which the separation of the particles is observed after centrifugation. With the SDS-PAGE (left) it can be seen that *PH*_*(1–110)*_ *GFP particles* highly pure particles are recovered in each gradient. **b** Confocal microscopy shows the presence of *PH*_*(1–110)*_ *GFP particles* of different sizes obtained in each sucrose gradient. **c** With the NTA equipment the sizes of the particles and the concentration of each particle were measured by 1 mL of solution. The peaks of the curves show the populations of particles. Dotted lines indicate the SD

We utilized a sucrose gradient to separate particles of different sizes (micro and nanoparticles). Indeed the *PH*_*(1–110)*_ *GFP particles* are polydisperse. A Coomassie Blue Staining from the SDS-PAGE shows a main protein component, corresponding to the molecular weight expected for *PH*_*(1–110)*_ *GFP* (Fig. 3a)*.* A sucrose gradient from 40 to 60% facilitates the separation of *PH*_*(1–110)*_ *GFP particles* of different sizes, which are evident when subjected to confocal microscopy imaging (Fig. 3b). Because some of the particles are smaller than the light diffraction limit of light microscopy, we conducted an additional analysis using nanoparticle tracking analysis (NTA, Methods). This analysis method clearly evidenced the multiple sizes in particles with the most abundant particles at around 115 nm (Fig. 3c).

### *PH*_*(1–110)*_*particles* produce a robust immune response in mice without adjuvant

**Fig. 4:**
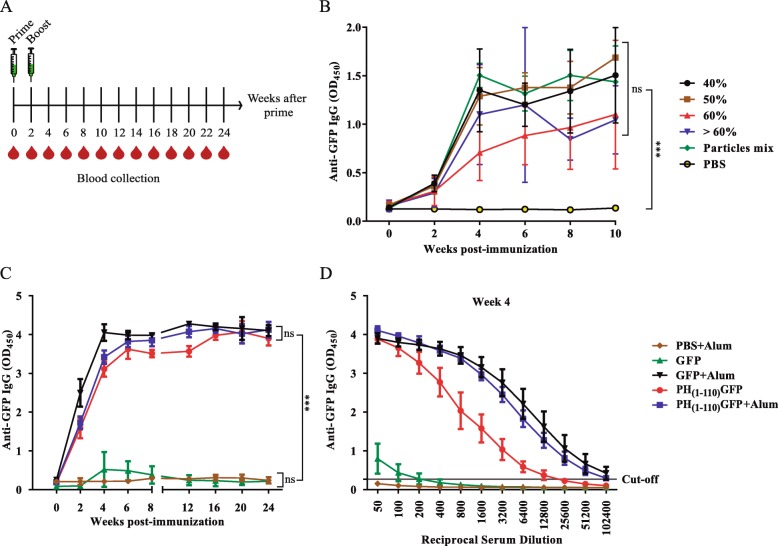
*The PH*_*(1–110)*_ *GFP particles induce immune response against GFP without the use of adjuvant.* **a** Immunization scheme of mice in which two immunizations are included on day 0 and 15, the blood sample was taken for 24 weeks at 15-day intervals. **b** First evaluation of the immune response induced by *PH*_*(1–110)*_ *GFP particles* of different sizes obtained in the sucrose gradients. In the immunized mice the production of IgG against GFP was measured by ELISA. All groups were compared with the particles mix group at week 10. **c** The antibody response against GFP was compared between particles mix of *PH*_*(1–110)*_ *GFP* with and without Alum and free GFP with and without Alum. **d** Serial 2-fold dilution of the sera of the mice to evaluate the antibody titers on week 4. The gray line indicates the cut-off. Error bars indicate the means ± SD (*n* = 5). *** *p* < 0.001, ns = not significant. (For panel B, two-way ANOVA with Dunnett post-tests and for panel C was used two-way ANOVA with Tukey post-tests)

Using the *PH*_*(1–110)*_ *GFP particles* we immunized mice to evaluate the antibody response generated by our particles. Initially we explored if one or two immunization would make a difference in the immune response and found no significant differences (see the Additional file 3). The immunization protocol included 2 vaccinations a week apart (Fig. 4a). Blood samples were taken in two-week intervals for 24 weeks to assay for antibodies against GFP used as model antigen (Fig. 4a). GFP is poorly immunogenic and adjuvants are required in order to obtain antibodies when using soluble GFP as antigen. Most interestingly, ELISA assays show the generation of anti-GFP antibodies in all animals vaccinated with the *PH*_*(1–110)*_ *GFP particles* obtained from the sucrose gradients shown in Fig. 3. As indicated by the data, no significant differences in the antibody generation were observed with any of the particles obtained from the 40–60% sucrose gradients (Fig. 4b). When the different particle sizes where compared with the combined mixture (particle mix), no statistically significant differences in antibody levels were observed. Thus, particle size does not appear to influence in the production of immunoglobulin IgG. Therefore, in the subsequent experiments we use the mixture of particles. The presence of anti-GFP antibodies was observed even after 24 weeks, indicating the induction of a long lasting immune response by the *PH*_*(1–110)*_ *GFP particles* (Fig. 4c). The antibody titers obtained with our *PH*_*(1–110)*_ *GFP particles* were high (12,800 dilution) without the use of any adjuvant. Using the gold standard adjuvant aluminum hydroxide (Alum) resulted in higher antibody titers (51,200, Fig. 4d). High antibody titers were maintained for at least 24 weeks post-vaccination (see the Additional file 4). Most noticeable, when GFP was used in the absence of any adjuvant, no antibodies were produced (Fig. 4c-d, green triangles). These results show that even though the use of adjuvant improves the immune response, the *PH*_*(1–110)*_ *GFP particles* can induce a robust, long lasting immune response comparable to that obtained with the use of an adjuvant (Fig. 4c-d). Thus, our results indicate that particles have an adjuvant effect, since using free GFP (without the particles) requires adjuvant in order to induce a measurable immune response (Fig. 4c-d).

Furthermore, we evaluated if the vaccination with *PH*_*(1–110)*_ *GFP particles* may induce immunity memory. Using a protocol to determine if subsequent exposure to the antigen may reactivate the immune response clearly showed that the original vaccination with *PH*_*(1–110)*_ *GFP particles* induce long lasting immune memory (see the Additional file 5).

### The immune response induced by *PH*_*(1–110)*_*particles* is both cellular and humoral

**Fig. 5:**
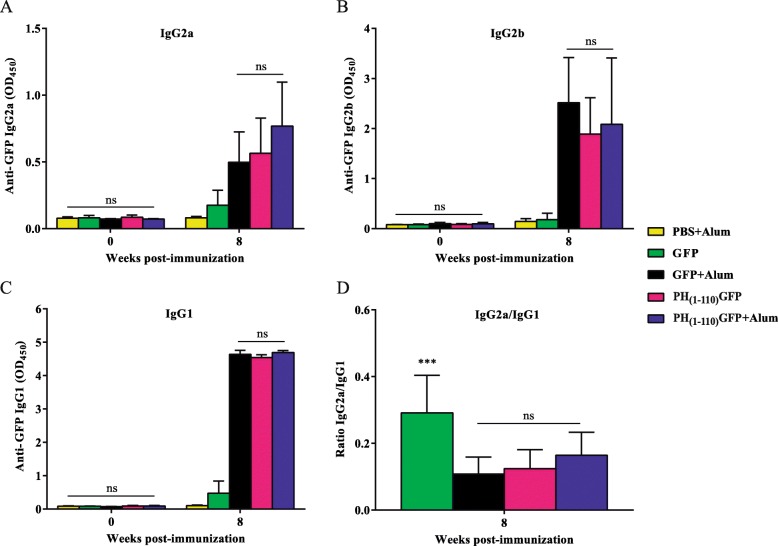
*The antibody response to GFP induced by the polyehedrin particles suggests a Th1 and Th2 profile.* After 8 weeks when the immune response was established immunoglobulin G subtypes were evaluated by ELISA; **a** IgG2a, **b** IgG2b and **c** IgG1 showing no significant difference between the groups: GFP + Alum, *PH*_*(1–110)*_ *GFP* and *PH*_*(1–110)*_ *GFP* + Alum, but these groups had at least a *p*-value < 0.05 against the PBS + Alum and GFP groups. **d** The IgG2a/IgG1 ratio was obtained to determine the type of predominant profile (Th1 or Th2) of the immune response generated by the *PH*_*(1–110)*_ *GFP particles*. Error bars indicate the means ± SD (*n* = 5). *** *p* < 0.001; ns = not significant. (Two-way ANOVA with Tukey post-tests)

Comparing pre-immune sera with sera obtained from mice after 8 weeks post vaccination we observed high levels of immunoglobulin IgG2a (Fig. 5a) and IgG2b (Fig. 5b) as well as IgG1 (Fig. 5c). The IgG2a/ IgG1 ratio obtained suggest a Th1 and Th2 mixed response, strongly suggesting that the *PH*_*(1–110)*_ *GFP particles* may induce the production of antibodies and moderate phagocytic activity (Fig. 5d). To further confirm that the vaccination with *PH*_*(1–110)*_ *GFP* particles induce also cellular immunity, we conducted a cell proliferation experiment (see the Additional file 6) comparing the *PH*_*(1–110)*_ *GFP particles* against Freund’s adjuvant that generates a strong cellular response [31]. As illustrated in the figure, the *PH*_*(1–110)*_ *GFP particles* induced a moderate cellular proliferation, characteristic of cellular immunity.

These results indicate that the *PH*_*(1–110)*_ *GFP particles* induce both humoral (antibodies) and cellular immunity.

### *PH*_*(1–110)*_*particles* are stable for over 1 year at ambient temperature

**Fig. 6:**
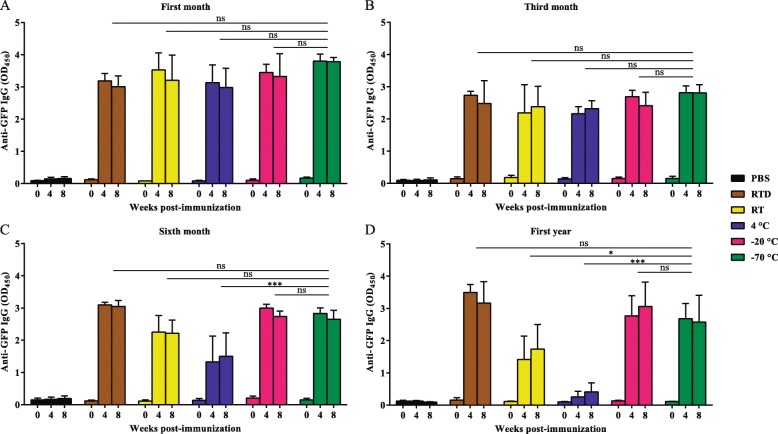
*The PH*_*(1–110)*_ *GFP particles are stable after 1 year at room temperature. PH*_*(1–110)*_ *GFP particles* that were maintained under different temperature conditions during: **a** 1 month, **b** 3 months, **c** 6 months and **d** 1 year were injected into mice and IgG antibodies specific to GFP were measured. All groups were compared against the group of mice immunized with particles maintained at − 70 °C. RTD = Room Temperature Dehydrated, RT = Room Temperature. Error bars indicate the means ± SD (*n* = 5). * *p* < 0.05; ** *p* < 0.01; *** *p* < 0.001; ns = not significant. (Two-way ANOVA with Dunnett post-tests)

Vaccines and drugs are usually stored at 4 °C or frozen, for this reason we designed a protocol to maintain *PH*_*(1–110)*_ *GFP* particles for up to a year under the following conditions: 1) stored at − 70 °C, 2) at − 20 °C, 3) at 4 °C, 4) at room temperature in solution and 5) at room temperature as dry powder. Every month during a year an aliquot of *PH*_*(1–110)*_ *GFP particles* stored at the different conditions specified above were used to immunize mice. The immune response was evaluated during the entire year by ELISA analysis of anti-GFP antibodies present in the sera from immunized animals. As illustrated in Fig. 6, storing the *PH*_*(1–110)*_ *GFP particles* at 4 °C impacted negatively its ability to generate antibodies after 6 months and more evidently after 1 year of storage. Most interestingly, keeping the *PH*_*(1–110)*_ *GFP particles* at room temperature as dry powder (R.T.D.) maintained the efficacy of the particles to induce a robust immune response (Fig. 6a-d). Similar results were obtained with *PH*_*(1–110)*_ *GFP particles* stored at − 20 °C and − 70 °C. These results indicate that storing the *PH*_*(1–110)*_ *GFP particles* as dry powder is equivalent to storing the particles at − 70 °C, since its ability to induce a robust immune response is not compromised after a year of storage.

## Discussion

The use of polyhedrin protein as a biotechnological tool has increased mainly due to the intrinsic capacity of self-aggregation [27]. In addition, we have shown that using only the first 110 amino acids of polyhedrin (*PH*_*(1–110)*_) retains the self-aggregation property [28]. The fusion protein spontaneously forms particles ranging in size from 100 nm to 1 μm. Unlike the particles formed by wild type polyhedrin which show a uniform size of around 1–2 μm [28], particles formed by the peptide *PH*_*(1–110)*_ are polydisperse (Fig. 3). Also the geometry of the wild type particles are polyhedral (hence the name polyhedra) while the particles produced by the peptide *PH*_*(1–110)*_ are irregular.

Recently, this property has been used for biomedical purposes to incorporate antigens into particles for vaccine development [29]. However, the thermostability of the particles formed has not been characterized until now. In the present study we use GFP (a poorly immunogenic protein) to characterize by confocal microscopy the particles formed by the fusion protein *PH*_*(1–110)*_ *GFP.*

Our experiments provide data on the physical nature of the particles formed. The wild type polyhedrin forms crystals of polyhedra, as demonstrated elsewhere [32]. Recently the crystal structure of wild type AcMNPV polyhedra has been elucidated using X-ray crystallography with a 3 Å resolution [33]. However, there are no studies aimed to determine the nature of the particles formed by *PH*_*(1–110)*_*.* This is important because it could help determine in later studies the degradation time of the *PH*_*(1–110)*_ particles in vivo. We observed a slow recovery after FRAP in *PH*_*(1–110)*_ *GFP particles* (Fig. 2) suggesting that the particles may form a dense structure similar to a liquid crystal [34]. Diffusion coefficients of proteins in agarose gel have been experimentally determined in dozens of hours [30].

One of the main problems in modern vaccines is the costly and lengthily purification process [10]. For this reason, the first step was to obtain a highly pure antigen easily and quickly. Because the *PH*_*(1–110)*_ *GFP particles* are insoluble in aqueous solutions, their purification is simple involving a one-step centrifugation process at low speed [28, 29].

By analyzing the type of immunoglobulins produced during an immune response, one can estimate if the response is T helper type 1 (Th1) or type 2 (Th2). Th1 lymphocytes stimulate Th1 immunity, which is characterized by the production of IFN-γ cytokine; while Th2 cells stimulate type 2 immunity, characterized by high antibody levels [35, 36]. A mixed response is desired in vaccines, which gives greater effectiveness in preventing diseases [35]. In the sera of mice immunized with our *PH*_*(1–110)*_ *GFP particles* we observed the stimulation of both the Th1 and Th2 response, in addition, high antibody and long duration titers were observed. The immuniglobulin profile clearly shows the mixed response, which is consistent with the lymphoproliferation analysis. However, this study was not aimed to an exhaustive analysis of the immune response, which can include the evaluation of subpopulations of T lymphocytes as well as cytokines induced by the particles.

On the other hand, an important finding was the adjuvant effect of the *PH*_*(1–110)*_ peptide. Adjuvants are primarily designed to improve the presentation of antigens, increase the immune response, as well as reduce doses [4]. In *PH*_*(1–110)*_ *GFP particles* a robust immune response was observed without the need for adjuvant. We found antibodies for the antigen (GFP) and for *PH*_*(1–110)*_ as expected. However, many adjuvants generate immune responses and.

antibodies against them. Several reports have found antibodies against adjuvants such as squalene [37]. This is a disadvantage for many adjuvants used in commercially available vaccines but does not appear to impair vaccine efficiency as all vaccines use adjuvants.

The use of only *PH*_*(1–110)*_ *GFP* particles without adjuvant achieved the same response as that achieved with aluminum hydroxide (Fig. 4c). The antibody titers remained high for more than 14 weeks. This finding highlights the adjuvant effect of the *PH*_*(1–110)*_ *GFP* particles*.*

It has been observed that the size of some particles affects the immune response [38, 39]. *PH*_*(1–110)*_ *GFP particles* of different sizes were evaluated without finding a difference in the response of immunoglubulin G.

Finally, the most relevant finding is the thermostability of *PH*_*(1–110)*_ *GFP particles*. An ideal vaccine should be also thermostable to avoid the so-called “cold chain”, which represents approximately 80% of the price of modern vaccines [17, 18]. There are biomaterials that have shown thermostability, however, few have shown stability after 1 year at room temperature [23]. Our result strongly suggest that the *PH*_*(1–110)*_ fragment retains the capacity to preserve proteins found in the wild type polyhedra [24, 25]. Interestingly, storing particles at 4 °C for more than 6 months compromised their ability to generate a robust immune response, suggesting that integrity of the antigen was compromised during storage at this temperature, which was confirmed by electrophoresis. This was not observed with particles stored at room temperature or frozen (− 20 °C or − 70 °C).

## Conclusion

We have developed a universal system to generate particles using peptides and proteins of interest as antigens. We coupled our method to the baculovirus expression system in order to generate large amounts of our fusion protein. Particles are purified by a single centrifugation step, showing purity higher than 80%. The particles are stable for at least 1 year at room temperature, preserving the antigenicity of the proteins of interest. This finding opens the possibility to significantly reduce the costs of conservation and distribution of vaccines.

Immunization with particles results in a robust humoral and cellular immunity. Antibody levels lasts for several months after vaccination with our particles in the absence of adjuvant.

## Methods

### Design of recombinant baculoviruses

For the generation of recombinant baculoviruses was used the expression vector pFastbac™1 of Bac-to-Bac® baculovirus expression system (Thermo Fisher, USA, cat. no.10359–016). Under the promoter of polyhedrin (*polh*), the genetic sequence of the first 330 bp of the N-terminal region of the polyhedrin was cloned and the genetic sequence of the GFP was ligated into its C-terminal in an open reading frame to generate a fusion protein called *PH*_*(1–110)*_ *GFP* [28]. The *polh* promoter and the polyhedrin sequence were taken from *Autographa californica multiple nucleopolyhedrovirus* virus (AcMNPV). For the generation of the *PH-WT-GFP* chimeric polyhedra, the pFastbac™ Dual expression vector (Thermo Fisher, USA, cat. No.10712024) was used, the WT polyhedrin was cloned under the *p10* promoter and the *PH*_*(1–110)*_ *GFP* under *polh* promoter. The baculoviruses were amplified, purified and titrated by following the recommendations and protocols provided by the supplier (Thermo Fisher, USA).

### Cell line and recombinant baculovirus

To propagate the recombinant baculoviruses and titrate them we used the *Spodoptera frugiperda* cell line, Sf9 (ATCC®, USA, cat. no. CRL-1711). Cells were maintained in Grace’s medium (Thermo Fisher, USA, cat. no. 11300–027) supplemented with 10% inactivated fetal bovine serum (FBS) (Biowest, France, cat. no. S1650–500), lactoalbumin (Sigma-Aldrich, USA, cat. no. 19010), yeastolate (Thermo Fisher, USA, cat. no. 292805), antibiotic-antimycotic (Thermo Fisher, USA, cat. no. 15240–062) and 0.1% pluronic acid F- 68 (Sigma-Aldrich, USA, cat. no. P1300) at 27 °C under agitation, as previously described [28].

### Production and purification of *PH*_*(1–110)*_*GFP particles*

SF9 cells (2 × 10^6^ cel/ml) were infected using a multiplicity of infection (moi) of 10 with the recombinant baculoviruses, the cells were maintained at 27 °C under agitation at 100 RPM, 72 h post infection (hpi) the cultures were centrifuged at 4200 g for 15 min to recover the viruses and obtain the cell pellet. The pellets were resuspended in phosphate buffered saline (PBS, 137 mM NaCl, 2.7 mM KCl, 10 mM Na_2_HPO_4_, 2 mM KH_2_PO_4_, pH 7.4) and were sonicated with 5 cycles of 20 s per pulse with 30% amplitude (Qsonica 700, USA). Between each cycle were maintained on ice for 5 min. After the last cycle, the *PH*_*(1–110)*_ *GFP particles* were washed 5 times with PBS, between each wash the samples were centrifuged at 14,000 g. Finally, they were resuspended in PBS. In addition, chimeric polyhedra were generated by infecting SF9 cells with baculovirus with the WT polyhedrin and recombinant polyhedrin *PH*_*(1–110)*_ *GFP*.

### Separation of *PH*_*(1–110)*_*GFP particles* by sucrose gradients

The *PH*_*(1–110)*_ *GFP particles* were separated in a discontinuous gradient of sucrose. To form the discontinuous gradient, 3 different sucrose concentrations were used, 40, 50 and 60% (w/v) in distilled water, ultracentrifugation was performed at 17,738 g (SW 40 ti rotor, Beckman Coulter, USA) for 10 min at 4 °C. The *PH*_*(1–110)*_ *GFP particles* of the different gradients were recovered and 3 washes were carried out with PBS, centrifuging the samples at 14,000 g after each wash.

### Protein quantification

The total protein of the lysates and the particles of the different gradients was determined using the Pierce™ BCA Protein Assay Kit (Thermo Fisher, USA, cat. no. 23225) based on bicinchoninic acid (BCA) for colorimetric detection.

### Cell confocal microscopy

SF9 cells infected with recombinant baculoviruses, 72 hpi were washed with PBS and incubated for 5 min with DAPI (4′, 6-diamino-2-phenylindole) to mark the nucleus (Thermo Fisher, USA, cat. no. D3571) at a 1:1000 dilution and fixed in slide glass (76 × 26 mm) with DAKO Fluorescent Mounting Medium (Agilent, USA, cat. no. S3023) [28]. The GFP of the *PH*_*(1–110)*_ *GFP particles* was excited at 473 and DAPI was excited at 405 nm. Fluorescence emission was collected at 510 nm for GFP and 420 nm for DAPI. All images were taken with a Fluoview FV10i confocal microscope (Olympus®, Japan), using the 60 × NA 1.35 oil immersion objective (UPLSAPO60XO). The images were analyzed with the software, FV10ASW.

### *PH*_*(1–110)*_*GFP particles* confocal microscopy and 3D reconstruction

The purified *PH*_*(1–110)*_ *GFP particles* were fixed with DAKO Fluorescent Mounting Medium in glass slides (76 × 26 mm). To obtain the images, we used a wide-field inverted IX81 Olympus® microscope with 60 × 1.42 NA oil immersion objective, to MT-20 illumination system and EMCCD camera iXon-897 (Andor Technology South Windsor, CT, USA). The used excitation and emission filters were 470 and 520 nm/40 bandpass respectively. The images were analyzed using ImageJ software. Imaris software was used for the 3D recostruction of confocal images (Additional file 1).

### Transmission electron microscopy (TEM)

SF9 cells infected with recombinant baculovirus *PH*_*(1–110)*_ *GFP* were centrifuged, the pellet was washed with cacodylate buffer (0.08 M, pH 7.4) and fixed with 0.6% glutaraldehyde and 0.4% paraformaldehyde in cacodylate buffer for 10 min. Post-fixation was made with 1% osmium tetroxide in cacodylate buffer. The cells were included in an epoxy resin and cuts of 90 nm thickness were made. Then the samples were contrasted with uranyl acetate 1% for 10 min and with lead citrate for 2.5 min. The JEOL JEM 12,000 EXII microscope at 80 kV (Jeol USA, USA) was used to observe the samples.

### Scanning electron microscopy (SEM)

Briefly, the particles were purified and fixed with 2.5% glutaraldehyde in phosphate buffer (0.1 M, pH 7.4). Post-fixation was performed with 1% osmium tetroxide in phosphate buffer. The samples were dehydrated with alcohol gradients and dried to critical point and coated with gold for observation. Finally, the JEOL JSM 5410LV microscope (Jeol USA, USA) was used to observe the samples.

### FRAP experiments

We peformed Fluorescence Recovery After Photobleaching (FRAP) using a Zeiss LSM 780 scanning confocal microscope (Axio observer. Z1/7) with an objective Plan-Apochromat 63 × / 1.40 oil DIC M27 (Carl Zeiss, Germany). The photobleaching protocol consisted in exposing the circular region-of-interest (ROI) to 488 nm Ar + laser at 100% of relative intensity in each *PH*_*(1–110)*_ *GFP particles*. The photobleaching lasted for approximately 1–2 s, and the fluorescence intensity images after photobleaching were collected at intervals of 4 min during 2 h, resolution using a pinhole of 40.96 μm. Detection wavelength was at 510 nm. Laser intensity settings of 1% were sufficient to illuminate the fluorescent label without causing significant photobleaching. The images were analyzed with ZEN 2012 software (blue edition, Carl Zeiss, Germany) and the final images were edited with ImageJ 1.52n (NIH, USA). We compared the recovery of fluorescence between the *PH*_*(1–110)*_ *GFP particles* (*n* = 14) and the chimeric particles *PH-WT-GFP* (*n* = 10) at different times. For this experiment, the *PH*_*(1–110)*_ *GFP particles* and the *PH-WT-GFP* particles were prepared in the same way as for confocal microscopy.

### Protein electrophoresis

Fresh *PH*_*(1–110)*_ *GFP particles* or *PH*_*(1–110)*_ *GFP particles* recovered from the different gradients of sucrose and GFP (Merck Millipore, USA, cat. no. 14–392) were mixed with 5 × Laemmli buffer (50 mM Tris-HCL, 3% SDS, 1% β-mercaptoethanol, 20% glycerol, 0.7% bromophenol blue, pH 6.8). The proteins were separated by 12% SDS-polyacrylamide gel electrophoresis (SDS-PAGE) at 85 V for 2 h and stained using Coomassie brilliant blue R-250.

### Western blot

For western blot analysis, proteins contained in the SDS-PAGE were transferred to a nitrocellulose membrane (Merck Millipore, USA, cat. no HATF00010) at 100 V for 1 h in wet chamber using transfer buffer (48 mM Tris base, 39 mM glycine, 0.037% SDS, 20% methanol). Membrane was blocked with 5% fat-free milk in Tris-buffered saline (TBS, 50 mM Tris-Cl, pH 7.6, 150 mM NaCl) over night (ON). The anti-GFP antibody utilized in these studies was produced in mice in our laboratory. The antibody was used at a 1:2000 dilution in TBS-T (0.05% Tween) and 0.5% fat-free milk. Membranes were incubated with anti-GFP antibody for 3 h with agitation at room temperature (RT). The secondary antibody was horseradish peroxidase-coupled (HRP) anti-mouse IgG (Sigma-Aldrich, USA, cat. no. A9044) was used at dilution 1:5000 in TBS-T and 0.5% fat-free milk. The secondary antibody was incubated 1 h in agitation at RT. The membranes were analyzed with a C-Digit Blot scanner (LI-COR, USA) and the signal generated by the SuperSignal® West Femto substrate (Thermo Fisher, USA, cat. no. 34095) was taken using the Image Studio software.

### Purity and conservation of *PH*_*(1–110)*_*GFP particles*

The purity of fresh *PH*_*(1–110)*_ *GFP particles* (Additional file 2) was evaluated by run electrophoretic assays using the Agilent Bioanalyzer 2100 (Agilent Technologies, USA) equipped with the Protein 230 assay kit according to the manufacturer’s recommended protocol. The electropherograms and gel-like images results were analyzed with Agilent 2100 expert software (Agilent technologies, USA).

### Nanoparticle tracking analysis (NTA)

The NanoSight instrument (Malvern Panalytical, UK) was used to determine the size of the polyhedrin particles produced by the recombinant baculoviruses. The *PH*_*(1–110)*_ *GFP particles* resuspended in sterile water were injected in a volume of 1 ml into the sample chamber. Five readings were made for each sample processed to obtain the average particle sizes. The NanoSight software (Malvern Panalytical, UK) tracked the Brownian motion in real-time to determine the center of the *PH*_*(1–110)*_ *GFP particles* and determine the diffusion coefficient of each particle. Finally, the software based on the Stokes-Einstein equation calculated the size of the particles [40, 41].

### Animal studies

All animals were provided by the bioterium of the Institute of Cellular Physiology. For the care, feeding, management and euthanasia of the animals, we followed the guidelines established by the Official Mexican Standard NOM-062-ZOO-1999, by the Institutional Subcommittee for the Care and Use of Experimental Animals (SICUAE) of the Faculty of Veterinary Medicine and Zootechnics (Protocol number DC-2017/2–3) and by the Internal Committee for the Care and Use of Laboratory Animals (CICUAL) of the Institute of Cellular Physiology (Protocol number LVD102 (66)-16), both committees attached to the National Autonomous University of Mexico (UNAM).

### Immunization studies

Female BALB/c mice 6–8 weeks of age and 20–25 g in weight were used for all experiments, and were kept in groups of 5. All groups were randomly formed from approximately 3 litters. The groups were kept in cage with solid continuous walls and floors and removable grating cover in a pathogen-free environment. Animals were provided with water and food ad libitum, bed of sawdust, sterile cardboard rolls as environmental enrichment. The immunization route was intramuscular (i.m.). The treatments were suspended in PBS and all treatment groups received the dose of antigen on days 0 and 14, this was decided after the dose response assay (Additional file 3). The control groups were treated first, then the groups without adjuvant and finally the groups with adjuvant. Blood samples were collected from day 0 until the end of each study at 2-week intervals. Samples were centrifuged, and the sera were stored at − 70 °C until analysis by Enzyme Linked Immunosorbent Assay (ELISA). For euthanasia of the animals we used a CO_2_ chamber at concentration of 70% for 3 min. In each study, particular points are described.

### Dose response assay

Five groups (*n* = 5) were subjected to the following treatments: Group 1: *PH*_*(1–110)*_ *GFP* 25 μg (one dose); Group 2: *PH*_*(1–110)*_ *GFP* 25 μg (two doses); Group 3: *PH*_*(1–110)*_ *GFP* 100 μg (one dose); Group 4: *PH*_*(1–110)*_ *GFP* 100 μg (two doses); and Group 5: PBS (control group) (Additional file 3). Blood samples were taken at 2-week intervals for 6 months.

### *PH*_*(1–110)*_*GFP particles* vs aluminum hydroxide (Alum)

From the dose response assay the treatment with *PH*_*(1–110)*_ *GFP* 25 μg two doses for subsequent experiments was selected. The following groups (*n* = 5) were evaluated: Group 1: GFP 25 μg; Group 2: GFP 25 μg + Alum; Group 3: *PH*_*(1–110)*_ *GFP* 25 μg; Group 4: *PH*_*(1–110)*_ *GFP* 25 μg + Alum; and Group 5: PBS + Alum (control group). The dilution used for Alum was 1:1. Blood samples were collected at 2-week intervals. With the serum samples obtained, the immune response was measured over time and antibody titers were evaluated at weeks 4 (Fig. 4b), 14 and 24 (Additional file 4). To evaluate the Th1 and Th2 responses, total IgG, IgG1, IgG2a, and IgG2b were measured and the IgG2a/IgG1 ratio was calculated (Fig. 5).

### Long-lived antibody responses

In week 24th week of the experiment *PH*_*(1–110)*_ *GFP particles* vs aluminum hydroxide (Alum), all groups received a boost with 5 μg of free GFP without adjuvant. Serum samples were obtained at day 4, 7, 14 and 21 post-immunization (Additional file 5).

### Thermostability evaluation

Stocks of *PH*_*(1–110)*_ *GFP particles* were stored at different conditions: 1) Room Temperature Dehydrated (RTD); 2) Room Temperature (RT); 3) 4 °C; 4) -20 °C; and 5) -70 °C. After 1, 3, 6 and 12 months of maintaining the particles in the different conditions, stock of each condition was taken and 6 mice group (*n* = 5) including a control group (PBS) were immunized. The RTD particles were dehydrated using a vacufuge™ concentrator 5301 (Eppendorf, Germany, cat. no. 5301) at a centrifugal force of 240 g at 30 °C for 30 min and were resuspended in PBS before being injected. In this experiment, no adjuvant was used. Blood sampling was performed for 2 months at 2-week intervals.

### Immune response with *PH*_*(1–110)*_*GFP particles* of different sizes

With the particles purified by discontinuous gradient of sucrose, the following groups of mice were immunized (*n* = 5): Group 1: *PH*_*(1–110)*_ *GFP particles* gradient 40%; Group 2: *PH*_*(1–110)*_ *GFP particles* gradient 50%; Group 3: *PH*_*(1–110)*_ *GFP particles* gradient 60%; Group 4: *PH*_*(1–110)*_ *GFP particles* gradient > 60%; Group 5: *PH*_*(1–110)*_ *GFP particles* gradients mixture; and Group 6: PBS. All treatments were conducted without adjuvant. Blood samples were collected for 10 weeks every 14 days.

### Immunization for proliferation assay

For this assay, 3 groups of mice were immunized (*n* = 5): Group 1: *PH*_*(1–110)*_ *GFP particles* 25 μg: Groups 2: *PH*_*(1–110)*_ *GFP particles* 25 μg + Adjuvant; and Group 3: PBS + Adjuvant. In this experiment the complete Freund’s adjuvant (CFA) (Sigma-Aldrich, USA, cat. no. F5881) and incomplete Freund’s adjuvant (IFA) (Sigma-Aldrich, USA, cat. no. F5506) were used. We decided to use the CFA and IFA in this experiment because, unlike Alum, broader stimulation of the cellular response has been previously observed [31, 42]. Blood samples were taken for 6 weeks at 14-day intervals (Additional file 6A).

### Lymphoproliferation assay

Mice were euthanized at week 6 post-immunization. Splenocytes were isolated from 3 animals from each treatment group by spleen perfusion with RPMI 1640 medium (Thermo Fisher, USA, cat. no. 31800022). Cells were treated and resuspended in RPMI 1640 supplemented medium and incubated with 5-(and-6) -Carboxyfluorescein Diacetate, Succinimidyl Ester (CFSE) (Thermo Fisher, USA, cat. no. C1157) as previously described [43]. Cells were stimulated with concanavalin A (ConA) (3 μg mL^− 1^) (data not shown), GFP (10 μg mL^− 1^), *PH*_*(1–110)*_ *GFP* (10 μg mL^− 1^) or Albumin (10 μg mL^− 1^, as a non-related antigen), and finally incubated in flat-bottomed microtiter plates (5 × 10^5^ cells/well), for 5 days at 37 °C in a 5% CO_2_ humidified atmosphere.

### Flow cytometry analysis

Cell proliferation was evaluated using standard flow cytometry protocols [43, 44]. After 5 days cells were harvested and stained with Phycoerythrin Cyanin 5.1 (PE-Cy™ 5)-conjugated anti-CD3 (BD Biosciences, USA, cat. no. 553065). T lymphocytes proliferation was determined by measuring the progressive loss of CFSE fluorescence within daughter cells in each cell division. Results were expressed as a percentage of proliferation (Additional file 6B). The cells were analyzed on the Attune® Acoustic Focusing Cytometer (blue/red system) using the Attune® Cytometric Software (Thermo Fisher, USA). At least 10,000 events were collected. The final analysis of the data was performed using FlowJo 7.6.2 software (FlowJo LLC, USA).

### Enzyme-linked immunosorbent assay (ELISA)

To determine the presence of GFP-specific antibodies in immunized mouse sera, samples were analyzed by ELISA. ELISA analysis was carried out using microtiter plates (Corning, USA, cat. no 3590) coated overnight with 50 μL of GFP at a concentration of 1 μg mL^− 1^ in 0.1 M sodium bicarbonate buffer (pH 9.2). Microplates were washed 5 times with 200 μL of PBS containing 0.2% Triton X-100 and blocked with PBS-Triton + 5% fat-free milk for 1 h at 37 °C. Then, 50 μL of the sera diluted 1:100 in PBS-Triton-fat-free milk (for the experiment of *PH*_*(1–110)*_ *GFP particles* of different sizes a dilution 1:400 was used) were added and plates were incubated 1 h at 37 °C. After washing as described above, 50 μL of anti-mouse IgG diluted 1:5000 (Sigma-Aldrich, USA, cat. no. A9044) or anti-mouse IgG1 diluted 1:3000 (Thermo Fisher, USA, cat. no. 04–6120) or anti-mouse IgG2a diluted 1:3000 (Abcam, UK, cat. no. ab98698) or anti-mouse IgG2b diluted 1:3000 (Thermo Fisher, USA, cat. no. 610320) (all HRP-conjugated) were added and plates were incubated 1 h at 37 °C. Plates were washed 5 times as described, 50 μL of the 3,3′,5,5′-Tetramethylbenzidine (TMB) substratum was added to each well (Sigma-Aldrich, USA, cat. no. 00–2023) and microplates were incubated at RT for 20 min. 50 μL of 0.16 M sulfuric acid solution was added to each well to stop the reaction. The OD reading at 450 nm was registered using Multiskan FC 3.1 microplate reader (Thermo Fisher, USA). For the titration of antibodies, sera were tested by performing serial 2-fold dilutions from 1:50 to 1:102400.

### Statistical analysis

All statistical analyses were performing using GraphPad Prism 7 software (GraphPad software, USA). Results were expressed as the means ± SD. All experiments were repeated at least once with comparable results. Data were analyzed by two-way ANOVA with a Tukey or Dunnett post-tests to correct for multiple comparison test. In the FRAP experiment to obtain the percentage of fluorescence recovery, the initial post-bleaching value (10 min) was subtracted from the last value obtained (140 min). To calculate FRAP differences was used in an unpaired, two-tailed Student’s t-test. To determine the cutoff in the titration of antibodies, the previously described methodology was used [45]. In the lymphoproliferation assay to obtain the absolute percentage of proliferation the PBS + Alum group value was subtracted from the other groups. A *p*-value < 0.05 was considered statistically significant. * *p* < 0.05; ** *p* < 0.01; *** *p* < 0.001 and ns = not significant.

## Supplementary information

**Additional file 1: Video S1.***3D reconstruction of PH*_*(1–110)*_*GFP particles within the nucleus of an insect cell.*

**Additional file 2. ***The purity of PH*_*(1–110)*_ *GFP particles is greater than 80%.* A. Show the gel run by the bioanalyzer equipment with the sample of *PH*_*(1–110)*_ *GFP particles*. B. The data obtained with the bioanalyzer were plotted to obtain the percentage of purity of the *PH*_*(1–110)*_ *GFP particles*. Error bar indicates the means ± SD (*n* = 3).

**Additional file 3. ***Doses of low concentration of polyhedrin particles have the same effect as high concentration doses.* A. Evaluation of specific antibodies to GFP produced by a single dose of *PH*_*(1–110)*_ *GFP particles* with low and high concentration. B. Comparison of the production of antibodies against GFP by double dose of *PH*_*(1–110)*_ *GFP particles* with a low concentration and a high concentration. Error bars indicate the means ± SD (*n* = 5). *** *p* < 0.001; ns = not significant. (Two-way ANOVA with Tukey post-tests).

**Additional file 4. ***High titers of antibodies induced by PH*_*(1–110)*_ *GFP particles are maintained for a long time.* A. Antibody titers are shown at week 14 post-immunization. B. Antibody titers are observed with the different treatments in week 24 post-immunization. The gray line shows the cut-off point to determine the antibody titer. Error bars indicate the means ± SD (*n* = 5).

**Additional file 5. ***PH*_*(1–110)*_ *GFP particles generate immunological memory.* A. Schedule of the process of immunization of mice and taking blood sample for 24 weeks, the “challenge” with free GFP in week 24 and blood collection for 21 days is also shown. In the red box, the weeks that served to evaluate the immunological memory are shown. B. Antibodies generated after the “challenge” were monitored for 21 days. The comparison was made against the PBS + Alum group. Error bars indicate the means ± SD (*n* = 5). * *p* < 0.05; ** *p* < 0.01; *** *p* < 0.001 (Two-way ANOVA with Dunnett post-tests).

**Additional file 6. ***The proliferation of T lymphocytes is induced by polyhedrin peptide (1–110).* A. Scheme showing the process that was followed to evaluate the proliferation of T lymphocytes by flow cytometry. B. The percentage of proliferation induced by the stimulus of three different treatments is shown in three groups of mice previously immunized with: PBS + FA, *PH*_*(1–110)*_ *GFP* and PH_(1–110)_ GFP + FA. FA = Freund’s adjuvant. Error bars indicate the means ± SD (*n* = 3). * *p* < 0.05; ** *p* < 0.01; *** *p* < 0.001 (Two-way ANOVA with Tukey post-tests).

## Data Availability

The datasets used and/or analyzed during the current study are available from the corresponding author on reasonable request.

## Abbreviations

AcMNPV: *Autographa californica multiple nucleopolyhedrovirus*
Alum: Aluminum hydroxide
BCA: Bicinchoninic acid
CFA: Complete Freund’s adjuvant
CFSE: 5-(and-6)-Carboxyfluorescein Diacetate, Succinimidyl Ester
CICUAL: Internal Committee for the Care and Use of Laboratory Animals
ConA: Concanavalin A
DAPI: 4′, 6-diamino-2-phenylindole
ELISA: Enzyme linked immunosorbent assay
FBS: Fetal bovine serum
FRAP: Fluorescence recovery after photobleaching
GFP: Green fluorescent protein
hpi: Hours post infection
HRP: Horseradish peroxidase-coupled
i.m.: Intramuscular
IFA: Incomplete Freund’s adjuvant
Ig: Immunoglobulin
moi: Multiplicity of infection
NPs: Nanoparticles
NTA: Nanoparticle tracking analysis
PBS: Phosphate buffered saline
PE-Cy™5: Phycoerythrin Cyanin 5.1
ROI: Region-of-interest
RT: Room temperature
RTD: Room Temperature Dehydrated
SDS-PAGE: SDS-polyacrylamide gel electrophoresis
SEM: Scanning electron microscopy
SICUAE: Institutional Subcommittee for the Care and Use of Experimental Animals
TBS: Tris-buffered saline
TEM: Transmission electron microscopy
Th1: T helper type 1
Th2: T helper type 2
TMB: 3,3′,5,5′-Tetramethylbenzidine
WHO: World Health Organization
WT: Wild type
