# Supplementary material for: A self-aggregating peptide: implications for the development of thermostable vaccine candidates
Source: BMC Biotechnol. 2020 Jan 21;20:1. doi: 10.1186/s12896-019-0592-9 (PMC6971912; doi:10.1186/s12896-019-0592-9)
Supplement: Supplementary file 2 — Additional file 2. The purity of PH(1–110) GFP particles is greater than 80%. A. Show the gel run by the bioanalyzer equipment with the sample of PH(1–110) GFP particles. B. The data obtained with the bioanalyzer were plotted to obtain the percentage of purity of the PH(1–110) GFP particles. Error bar indicates the means ± SD (n = 3). [file 12896_2019_592_MOESM2_ESM.pdf]

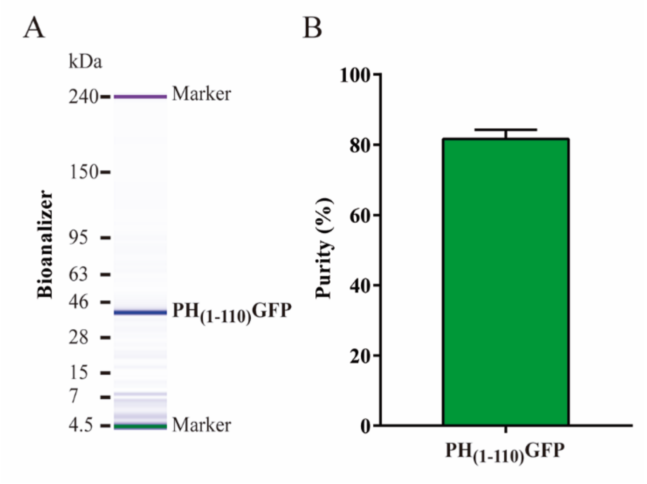

Additional file 2: ***The purity of PH$_{(1\text{-}110)}$GFP particles is greater than 80%.*** **A**. Show the gel run by the bioanalyzer equipment with the sample of *PH$_{(1\text{-}110)}$GFP particles*. **B**. The data obtained with the bioanalyzer were plotted to obtain the percentage of purity of the *PH$_{(1\text{-}110)}$GFP particles*. Error bar indicates the means ± SD (n = 3).
