# Supplementary material for: A self-aggregating peptide: implications for the development of thermostable vaccine candidates
Source: BMC Biotechnol. 2020 Jan 21;20:1. doi: 10.1186/s12896-019-0592-9 (PMC6971912; doi:10.1186/s12896-019-0592-9)
Supplement: Supplementary file 3 — Additional file 3. Doses of low concentration of polyhedrin particles have the same effect as high concentration doses. A. Evaluation of specific antibodies to GFP produced by a single dose of PH(1–110) GFP particles with low and high concentration. B. Comparison of the production of antibodies against GFP by double dose of PH(1–110) GFP particles with a low concentration and a high concentration. Error bars indicate the means ± SD (n = 5). *** p < 0.001; ns = not significant. (Two-way ANOVA with Tukey post-tests). [file 12896_2019_592_MOESM3_ESM.pdf]

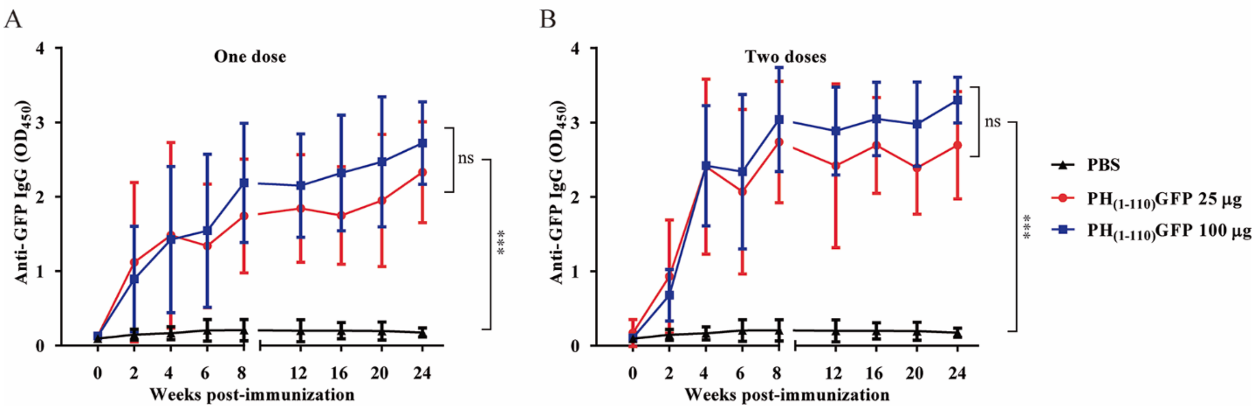

Additional file 3: ***Doses of low concentration of polyhedrin particles have the same effect as high concentration doses.*** **A**. Evaluation of specific antibodies to GFP produced by a single dose of *$PH_{(1\text{-}110)}GFP$ particles* with low and high concentration. **B**. Comparison of the production of antibodies against GFP by double dose of *$PH_{(1\text{-}110)}GFP$ particles* with a low concentration and a high concentration. Error bars indicate the means ± SD (n = 5). *** p <0.001; ns = not significant. (Two-way ANOVA with Tukey post-tests).
