# Supplementary material for: A self-aggregating peptide: implications for the development of thermostable vaccine candidates
Source: BMC Biotechnol. 2020 Jan 21;20:1. doi: 10.1186/s12896-019-0592-9 (PMC6971912; doi:10.1186/s12896-019-0592-9)
Supplement: Supplementary file 4 — Additional file 4. High titers of antibodies induced by PH(1–110) GFP particles are maintained for a long time. A. Antibody titers are shown at week 14 post-immunization. B. Antibody titers are observed with the different treatments in week 24 post-immunization. The gray line shows the cut-off point to determine the antibody titer. Error bars indicate the means ± SD (n = 5). [file 12896_2019_592_MOESM4_ESM.pdf]

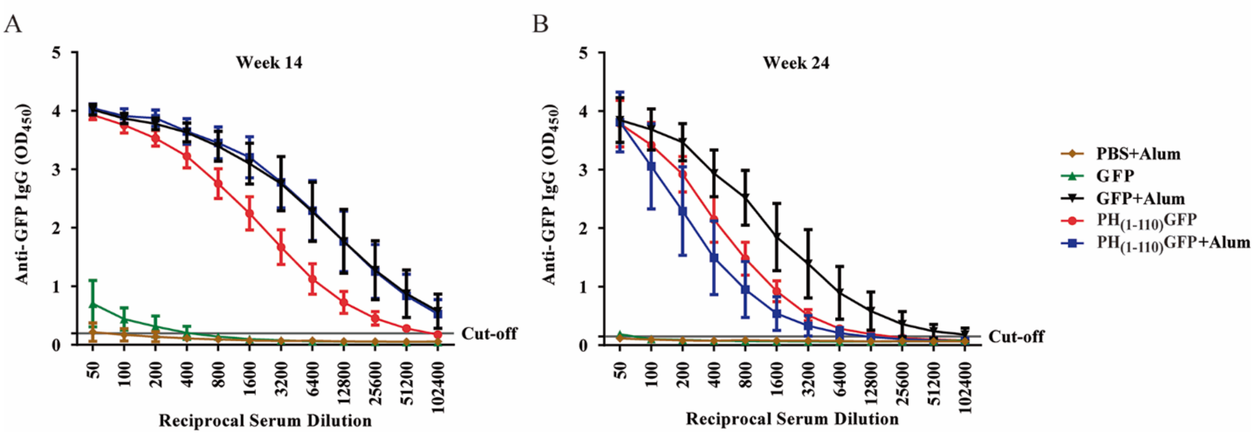

Additional file 4: ***High titers of antibodies induced by $PH_{(1\text{-}110)}$GFP particles are maintained for a long time.*** **A**. Antibody titers are shown at week 14 post-immunization. **B**. Antibody titers are observed with the different treatments in week 24 post-immunization. The gray line shows the cut-off point to determine the antibody titer. Error bars indicate the means ± SD (n = 5).
