# Supplementary material for: A self-aggregating peptide: implications for the development of thermostable vaccine candidates
Source: BMC Biotechnol. 2020 Jan 21;20:1. doi: 10.1186/s12896-019-0592-9 (PMC6971912; doi:10.1186/s12896-019-0592-9)
Supplement: Supplementary file 5 — Additional file 5. PH(1–110) GFP particles generate immunological memory. A. Schedule of the process of immunization of mice and taking blood sample for 24 weeks, the “challenge” with free GFP in week 24 and blood collection for 21 days is also shown. In the red box, the weeks that served to evaluate the immunological memory are shown. B. Antibodies generated after the “challenge” were monitored for 21 days. The comparison was made against the PBS + Alum group. Error bars indicate the means ± SD (n = 5). * p < 0.05; ** p < 0.01; *** p < 0.001 (Two-way ANOVA with Dunnett post-tests). [file 12896_2019_592_MOESM5_ESM.pdf]

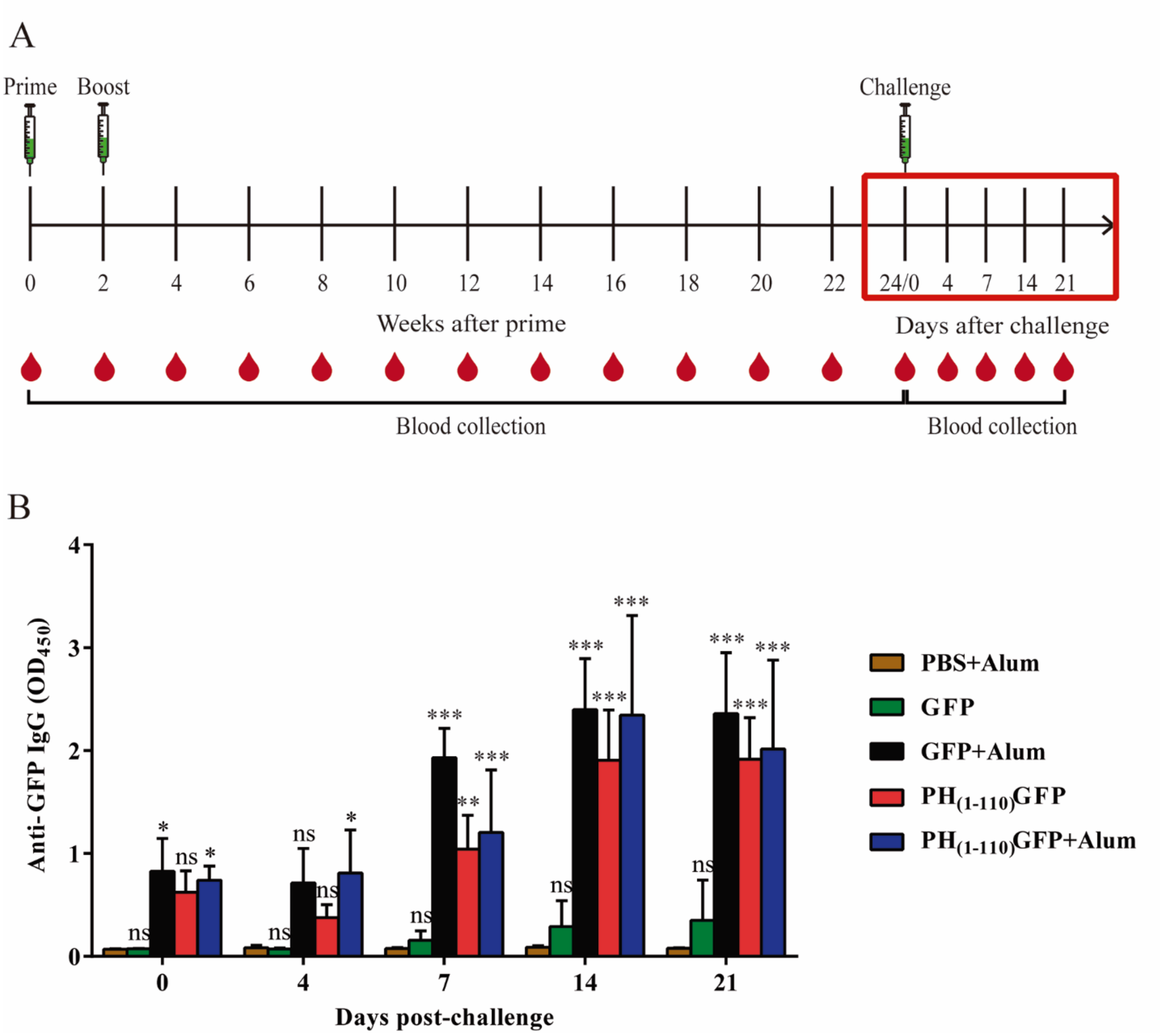

Additional file 5: ***PH$_{(1-110)}$GFP particles generate immunological memory.*** **A**. Schedule of the process of immunization of mice and taking blood sample for 24 weeks, the "challenge" with free GFP in week 24 and blood collection for 21 days is also shown. In the red box, the weeks that served to evaluate the immunological memory are shown. **B**. Antibodies generated after the "challenge" were monitored for 21 days. The comparison was made against the PBS+Alum group. Error bars indicate the means ± SD (n = 5). * p <0.05; ** p <0.01; *** p <0.001 (Two-way ANOVA with Dunnett post-tests).
