# Supplementary material for: A self-aggregating peptide: implications for the development of thermostable vaccine candidates
Source: BMC Biotechnol. 2020 Jan 21;20:1. doi: 10.1186/s12896-019-0592-9 (PMC6971912; doi:10.1186/s12896-019-0592-9)
Supplement: Supplementary file 6 — Additional file 6. The proliferation of T lymphocytes is induced by polyhedrin peptide (1–110). A. Scheme showing the process that was followed to evaluate the proliferation of T lymphocytes by flow cytometry. B. The percentage of proliferation induced by the stimulus of three different treatments is shown in three groups of mice previously immunized with: PBS + FA, PH(1–110) GFP and PH(1–110) GFP + FA. FA = Freund’s adjuvant. Error bars indicate the means ± SD (n = 3). * p < 0.05; ** p < 0.01; *** p < 0.001 (Two-way ANOVA with Tukey post-tests). [file 12896_2019_592_MOESM6_ESM.pdf]

A

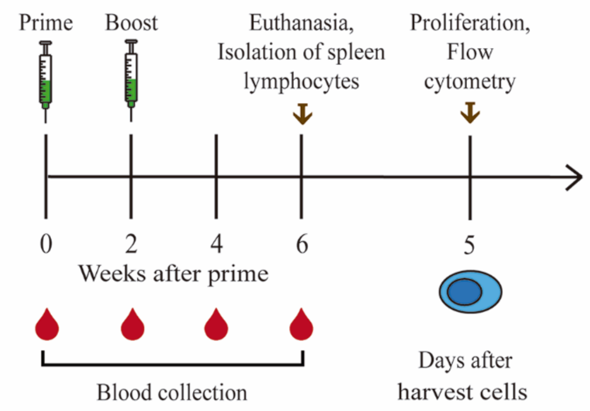

B

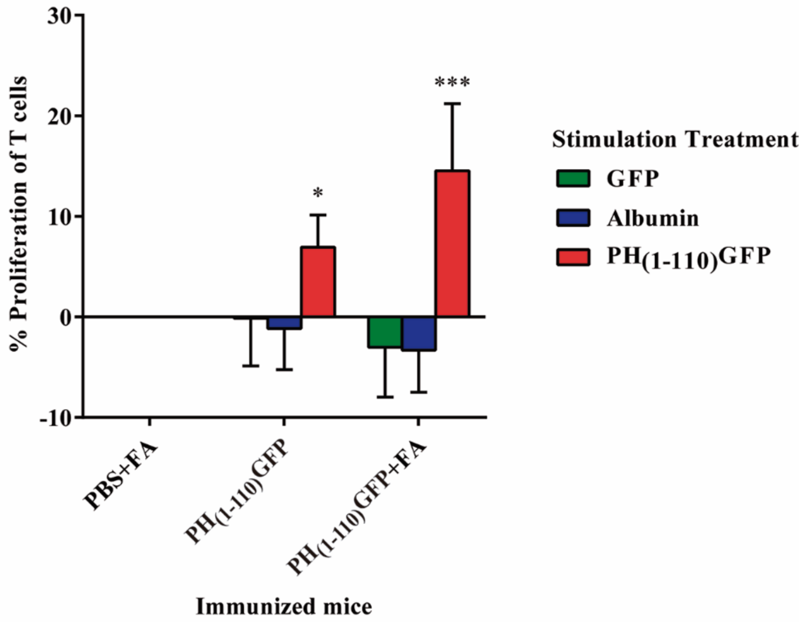

Additional file 6: ***The proliferation of T lymphocytes is induced by polyhedrin peptide (1-110).*** **A**. Scheme showing the process that was followed to evaluate the proliferation of T lymphocytes by flow cytometry. **B**. The percentage of proliferation induced by the stimulus of three different treatments is shown in three groups of mice previously immunized with: PBS+FA, *PH$_{(1-110)}$GFP* and PH$_{(1-110)}$GFP+FA. FA = Freund's adjuvant. Error bars indicate the means ± SD (n = 3). * p <0.05; ** p <0.01; *** p <0.001 (Two-way ANOVA with Tukey post-tests).
